# The false-negative rate of sentinel lymph node biopsy and its related factors in early-stage cervical cancer: a systematic review and meta-analysis

**DOI:** 10.2340/1651-226X.2026.44984

**Published:** 2026-03-16

**Authors:** Chao He, Fei Li, Min He, Jia Li

**Affiliations:** a Department of Obstetrics and Gynecology, Lequn Branch, The First Hospital of Jilin University, Changchun, Jilin Province, China; b Department of Hematology, The First Hospital of Jilin University, Changchun, Jilin Province, China

**Keywords:** Sentinel lymph node biopsy, uterine cervical neoplasms, systematic review, meta-analysis

## Abstract

**Background and purpose:**

The objective of this systematic review and meta-analysis was to evaluate the false-negative rate (FNR) of sentinel lymph node biopsy (SLNB) performed in patients with early-stage cervical cancer (ECC), and to study the risk factors affecting FNR.

**Material and methods:**

We searched three databases (Embase, MEDLINE, and Cochrane Central Library) for articles published in the last decade from January 2014 to September 2024. Publications on patients with ECC who underwent SLNB, with information on the FNR of SLNB, were included. The QUADAS-2 tool was used to assess the risk of bias and the clinical applicability of the included studies. The FNR and associated factors were synthesized using random-effects meta-analysis and meta-regression.

**Results:**

A total of 49 eligible studies with a low to moderate risk of bias were included in the final analysis. The overall FNR was 10.9% (95 CI: 6.0–16.7). No significant differences in FNR were found for different reference standards or tumor diameters (< 2 cm vs. ≥ 2 cm). However, different tracers (e.g. methylene blue [MB], carbon nanoparticle [CNP], indocyanine green [ICG], and Technetium-99m [Tc-99m] combined with other tracers) appear to account for the different FNRs. In the meta-regression analysis, we found that the proportion of SLNs located in the obturator area was significantly negatively associated with FNR (coefficient = −0.88, *p* = 0.04).

**Interpretation:**

The overall FNR of SLNB for ECC was approximately 10.9%. Factors that tended to reduce the FNR included using a low-volume metastatic detection technique, having a tumor diameter of < 2 cm, employing specific tracer regimens, and identifying more than one lymph node in the obturator fossa.

Registration: PROSPERO (CRD42024608411)

## Introduction

First published in 1992, sentinel lymph node biopsy (SLNB) is a procedure for identifying, removing, and analyzing a tumor’s first drainage lymph node [1, 2]. A negative SLNB indicates the possibility of no cancer metastasis in the surrounding lymph node pathways, based on the rationale that an SLN is the first stop in lymph node metastasis. Therefore, complete lymphadenectomy can be avoided, resulting in less surgical morbidity [3, 4].

Cervical cancer (CC) is one of the most common cancers and the leading cause of cancer-related deaths in women, with approximately 604,000 new cases and 342,000 deaths worldwide in 2020 [5]. Lymph node involvement is a significant prognostic factor in patients with early-stage cervical cancer (ECC), although it is present in less than 20% of patients at this stage [6, 7]. Extensive evidence has shown a good prognosis in terms of disease-free survival and overall survival after surgery for ECC [8–11]. SLNB was reported to be an excellent choice for the surgical treatment of ECC with less severe postoperative leg heaviness and fatigue, and a tendency toward a better quality of life when complete lymphadenectomy was avoided [12, 13]. Moreover, lymph node assessment is essential for determining the most appropriate therapeutic strategy for patients with ECC, since pelvic lymph node metastasis may need para-aortic lymphadenectomy and other adjuvant therapies rather than radical surgery [14, 15].

Studies of SLNB in ECC have established the procedure’s feasibility in this condition and have shown excellent detection rates and diagnostic value [16–18]. However, despite the advantages, the rate of missed metastatic lymph nodes remains a concern [19]. In practice, problems related to the false negative rate (FNR) for SLNB, including the significance of ultrastaging for prognosis, the choice of intraoperative tracers, and the validity of the frozen section (FS) diagnosis, remain controversial [19].

Although there are extensive articles examining the FNRs of SLNB for ECC, they are limited to reports of individual FNR rates. Few studies have examined risk factors for FNR in SLNB for ECC. The objective of this systematic review and meta-analysis was to evaluate the FNR of SLNB in patients with ECC at International Federation of Gynecology and Obstetrics (FIGO) stages IA to IIB [20] and to identify risk factors affecting FNR.

## Material and methods

This systematic review and meta-analysis followed the PRISMA 2020 statement [21]. The study protocol was registered in PROSPERO (CRD42024608411).

### Eligibility criteria

The inclusion criteria for the target articles included 10 or more women diagnosed with IA-IIB ECC according to the FIGO system [22]; study techniques using SLNB; after sentinel lymph node dissection; pelvic lymphadenectomy with or without para-aortic lymphadenectomy; permanent pathological examination was performed after intraoperative examination; and reported outcome metrics including but were not limited to FNRs.

The exclusion criteria were as follows: articles that did not meet the inclusion criteria; secondary lesions; repeat publications or non-original studies (e.g. systematic reviews and meta-analyses) and narrative reviews, abstracts, letters, editorials, and comments. The most recent study was included in the final analysis if duplicate datasets were present.

### Literature search

We searched three databases (Embase, MEDLINE, and Cochrane Central Library) for articles published within the last decade, from January 2014 to the date we performed the literature search (September 27, 2024). The key search terms included ‘Sentinel Lymph Node’, ‘Sentinel Lymph Node Biopsy’, ‘Uterine Cervical Neoplasms’, and different medical tracers. Details of the search strategies are provided in Supplementary Appendix 1. We decided to register this project before data extraction; therefore, the formal screening of search results against eligibility criteria began before submission to PROSPERO.

### Study selection

Two reviewers (FL and MH) independently selected studies for the two stages. The first stage involved the screening of the title and abstract. Two reviewers screened the records for eligibility, using EndNote 2021. Potentially eligible studies were selected after removing duplicates and verifying consistency across reviewers. The second stage was full-text screening. The full texts of the articles were downloaded for this screening stage using a pretested screening form. Reasons for excluding some articles were recorded. Any disagreement between the two reviewers was resolved by discussion or referral to a third party (CH).

### Data collection

Two reviewers (FL and MH) independently collected the data using a pre-tested data collection form (Supplementary Appendix 2) from November 30, 2024. Disagreements between the two reviewers during the data collection process were resolved through discussions with the research team. The collated data included the following: (1) the essential characteristics of the included studies, including the first author’s surname, publication year, country, study design, study period, demographic information of the target population (ECC), and potential factors related to the SLN test. (2) Details of the medical tracers used, FSs (yes/no), pathological examination methods (e.g. hematoxylin and eosin [H&E] and immunohistochemistry [IHC]), and the reference standard. (3) SLN data, including detection rate, SLN location, and FNR data.

### Outcomes

The primary outcome was the FNR of the SLNB, defined as the number of patients with lymph node metastasis without a positive SLN divided by all metastatic patients


$$\left(\frac{\text{The number of patients with false negative SLN}}{\text{The number of patients with true negative LN}}\right)$$

[23].

Secondary outcomes were the potential risk factors associated with SLNB FNR. The potentially related variables studied in this study included patient age (mean or median), body mass index (body mass index [BMI], mean or median), FIGO stage, histologic type (squamous cell carcinoma [SCC], adenocarcinoma [AD], adenosquamous cell carcinoma [AS] and other cancer histology types), lymphovascular space invasion (LVSI), previous conization (LEEP), tumor grade according to tumor risk (low risk = grade 1 to high risk = grade 3) [24], tumor diameter ≥ 2 cm, detection method (tracer strategies), neoadjuvant, reference standard, SLN location and SLN detection rate.

### Evaluation of risk of bias

Two reviewers (FL and MH) independently assessed the risk of bias (RoB) and clinical applicability for the studies included in the final analysis using the QUADAS-2 tool (Supplementary Appendix 3) [25]. Disagreements were resolved through discussion or referred to a third party (CH). The four domains (patient selection, index test, reference standard, and flow and timing) of the RoB and three components (patient selection, index test, and reference standard) of clinical applicability were evaluated accordingly. A RoB domain or a component of clinical applicability was classified as ‘low’, ‘unclear’, or ‘high’ risk of bias based on a set of signaling or evaluation questions for each unit. If all questions for a domain or component were answered as ‘yes’, then the risk of bias was rated as ‘low’; similarly, if any question was answered as ‘no’, then the risk of bias was rated as ‘high’. The ‘unclear’ category was assigned when insufficient information was available for that unit.

### Synthesis methods

All analyses were performed using a statistical software package (Stata version 12.0; Stata Corp., Texas, USA). Statistical significance was established at *p* < 0.05. Heterogeneity in the meta-analysis was assessed using the Q and I^2^ statistic. When *p* < 0.05, and *I*^2^ > 50%, the result was deemed heterogeneous, and a random-effects model was used for analysis. Otherwise, a fixed-effects model was used for meta-analysis [26, 27].

Meta-analysis of the FNR was performed using a random-effects model. Stratified meta-analysis and meta-regression were used to explore the impact of related variables (e.g. patient age, BMI, FIGO stage, histologic type, LVSI, LEEP status, tumor grade, tumor diameter, detection method, neoadjuvant therapy, reference standard, SLN location, and detection rate) on the pooled FNR results.

Sensitivity analysis was performed after excluding studies that did not use PLND as a reference standard. We used funnel plots and Egger’s test to assess publication bias [28, 29].

## Results

### Study characteristics

**Figure 1:**
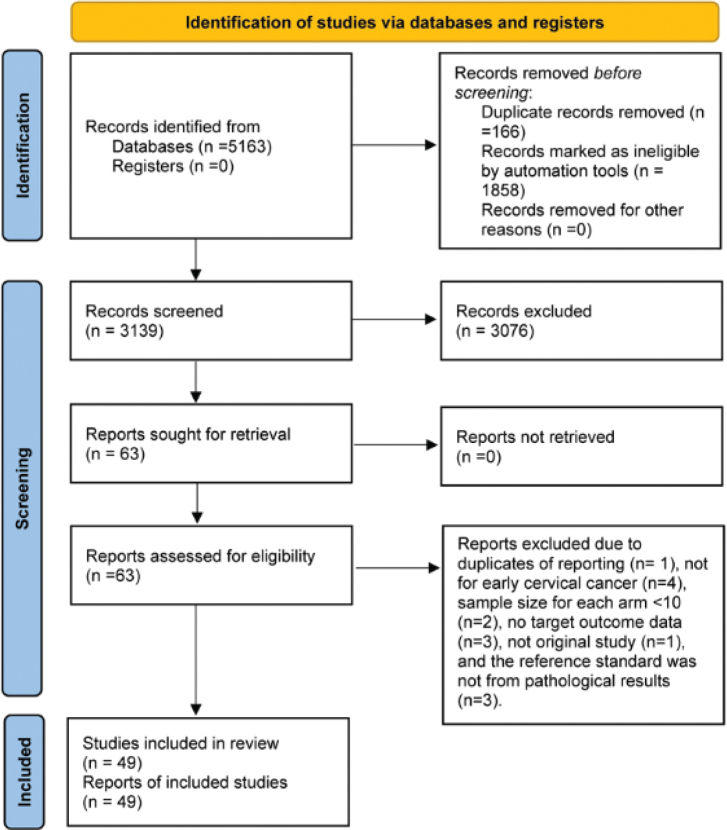
PRISMA flow diagram for the selection of articles for meta-analysis.

A total of 5,163 relevant studies were found by searching three databases (Medline, Embase, and Cochrane Central Library) from 2014 to 27 September 2024 (the search date). After excluding duplicates (*n* = 166) and records marked as ineligible by automation tools (*n* = 1,858), 3,139 remaining studies were screened for title and abstract. Next, the remaining 63 relevant studies underwent a full-text review. Studies that did not meet the study objectives were excluded. Finally, 49 studies [30–78] were included in the final analysis (Figure 1).

Of the 49 included studies, 28 (57.1%) were prospective. The total number of patients with ECC included was 5,004, with a range of 20–356 for individual studies. The median and mean ages were reported in 32 and 14 studies, respectively, except for three studies that did not specify the patients’ ages. The study reported ages ranging from 19 to 85 years old. The median BMI was reported in 30 studies with a range of 14.6 to 52.0 kg/m^2^. The most popular tracers and reference standards used were Technetium-99m (Tc-99m) ± other tracers (22/49, 44.9%) and pelvic lymph node dissection (PLND) (37/49, 75.5%), respectively (Table 1). Additionally, the FNR reported in SLNB studies ranged from 0 to 57.7%.

**Table 1:** Characteristics of the included articles.

Author and publication year (country)	Study design	Study period	Clinical stages (criteria)	Total sample size, *n*	Age (mean ± SD or specified), years	Tracer used	Reference standard
Zhang et al. 2014 (China) [30]	Prospective single-arm study	June 2009 to December 2010	IA2-IIA (FIGO 2008)	56	Median = 45.5 (range 23.0 to 67.0)	MB	Bilateral PLND
Bats et al. 2015 (France) [31]	Prospective multicenter study	January 2005 to June 2007	IA-IB1 (FIGO)	139	44.4 ± 13.6	Tc-99m+patent blue	Pelvic and para-aortic lymphadenectomy
de Freitas et al. 2015 (Brazil) [32]	Prospective longitudinal study	March 2008 to November 2010	IA2-IIA (FIGO)	57	Median = 42.0 (range 24.0 to 71.0)	Tc-99m+Patent blue	Systematic bilateral PLND
Imboden et al. 2015 (Switzerland) [33]	Prospective cohort study	April 2008 to January 2011	IA1-IIB (FIGO)	58	Mean = 47.0 for Patent blue group; Mean = 43.4 for ICG group	Tc-99m+Patent blue	Lymphadenectomy (based on the description of the paper)
Kato et al. 2015 (Japan) [34]	Retrospective analysis	January 2005 to December 2013	IB1 (Not specified)	102	Not specified	Tc-99m ± Patent blue ± ICG	lymphadenectomy
Buda et al. 2016 (Italy) [35]	Retrospective cohort study	October 2010 to May 2015	1A2–IB1 (Not specified)	45	Not specified	MB ± Tc-99m or ICG	Systematic PLND
Cibula et al. 2016 (Czech Republic) [36]	Prospective single-arm study	Not specified	IB1-IIB (Not specified)	17	Mean = 48.0; Median = 45.0 (range 32.0 to 69.0)	Tc-99m+blue dye	PLND
Cusimano et al. 2017 (Canada) [37]	Prospective, longitudinal cohort study	August 2010 to February 2014	IA1-IB1 (FIGO)	39	Median = 42.0 (range 28.0 to 61.0)	Tc-99m+patent blue	Bilateral PLND
Deng et al. 2017 (China) [38]	Prospective single-arm study	March 2003 to July 2015	IB1 (FIGO 2009)	49	Mean = 28.5 (range 19.0 to 40.0)	Tc-99m	Bilateral PLND
Di Martino et al. 2017 (Multiple European countries) [39]	Multicenter, retrospective observational study	January 2008 to December 2016	IB1-IIB (FIGO)	95	Median = 49.0 (range 26.0 to 77.0) for the Tc-99m group; Median = 46.0 (range 25.0 to 72.0) for the ICG group	Tc-99m+patent blue or ICG	Bilateral PLND
Lu et al. 2017 (China) [40]	Prospective single-arm study	January 2014 to January 2016	IA2–IIA (FIGO 2009)	40	Median = 42.0 (range 34.0 to 53.0)	CNP	Systemic PLND ± para-aortic lymphadenectomy
Papadia et al. 2017 (Switzerland) [41]	Retrospective analysis	December 2008 to November 2016	IA1–IIA (FIGO)	60	Median = 47.0 (range 27.0 to 72.0)	ICG or Tc-99m+patent blue	Bilateral PLND
Salvo et al. 2017 (United States) [42]	Retrospective analysis	August 1997 to October 2015	IA1–IIA1 (Not specified)	188	Median = 38.0 (range 21.0 to 68.0)	ICG, Tc-99m, patent blue, or Tc-99m+patent blue	Bilateral PLND
Tanaka et al. 2017 (Japan) [43]	Prospective cohort study	September 2012 to May 2016	IA-IIB (FIGO)	119	46.0 ± 10.7	Tc-99m, indigo carmine (IDC), or ICG	Systematic PLND
Buda et al. 2018 (Italy and Switzerland) [44]	Retrospective study	March 2011 to April 2017	IA-IB1 (FIGO 2009)	65	Median = 46.0 (range 29.0 to 71.0) for the Tc-99m group; Median = 42.0 (range 28.0 to 68.0) for the ICG group	Tc-99m ± patent blue	PLND
Cea Garcia et al. 2018 (Spain) [45]	Prospective single-arm study	January 2012 to April 2017	IA-IIA1 (FIGO)	23	46.0 ± 10.1	Tc-99m+MB	Bilateral PLND
Kim et al. 2018 (Republic of Korea) [46]	Single-center, retrospective study	August 2015 to January 2017	IA1-IIA (FIGO)	103	Median = 45.0 (range 29.0 to 77.0)	ICG	Bilateral PLND
Soergel et al. 2018 (Germany) [47]	Prospective single-arm study	May 2015 to March 2017	IA-IIB (FIGO)	33	Mean = 50.7 (range 33.0 to 82.0)	ICG or Tc-99m+patent blue	Complete PLND
Sonoda et al. 2018 (Japan) [48]	Retrospective study	June 2005 to May 2017.	In situ-IIA1 (FIGO)	201	Median = 33.0 (range 21.0 to 43.0)	Tc-99m	Permanent processing with final histological results of the SLN
Yahata et al. 2018 (Japan) [49]	Retrospective study	January 2009 to December 2015	IA-IIA1 (FIGO)	139	Median = 33.0 (range 21.0 to 73.0)	Tc-99m	Permanent processing with final histological results of the SLN
Diaz-Feijoo et al. 2019 (Spain) [50]	Prospective single-arm study	September 2000 to October 2016	IA2-IIA1 (FIGO 2009)	128	48.4 ± 12.2	Tc-99m+MB (or isosulfan blue)	Systematic and bilateral PLND
Balaya et al. 2020 (France) [51]	Prospective multicentric database study	January 2005 to December 2012	IA-IIA1 (FIGO 2018)	313	Median = 42.0 (range 22.0 to 85.0)	Tc-99m+patent blue	Permanent processing with final histological results of the SLN
Bizzarri et al. 2020 (Italy) [52]	Prospective cohort study	November 2017 to July 2019	IA1-IB1 (FIGO 2009)	18	Median = 40.5 (range 31.0 to 57.0)	ICG	Systematic PLND
Dostalek et al. 2020 (Czech Republic) [53]	Retrospective single-institution study	May 2005 to December 2015	CC with pT1a – pT2 (TNM stage)	309	44.4 ± 12.7	Tc-99m+patent blue	Systematic PLND
Favre et al. 2020 (France) [54]	Randomized multicenter trial	December 2008 to November 2011	IA1-IIA1 (FIGO 2009)	101	Median = 4 1.7	Tc-99m+patent blue	PLND
Gil-Ibanez et al. 2020 (Spain) [55]	Retrospective study	March 2005 to April 2018	IB1 (FIGO 2009)	19	Median = 33.5 (range 22.0 to 44.0)	Tc-99m+Isosulfan blue dye	Bilateral PLND
Luhrs et al. 2020 (Sweden) [56]	Prospective study	November 2014 to March 2017	IA-IIA (FIGO 2009)	65	Median = 39.0 (range 23.0 to 79.0)	Tc-99m, ICG, or ICG+Tc-99m	PLND
Papathemelis et al. 2020 (Germany) [57]	Retrospective single-arm, single-center study	December 2015 to April 2018	IA1-IIB (FIGO)	20	Mean = 51.2 (range 35.0 to 75.0)	ICG	Systematic PLND
Rychlik et al. 2020 (France) [58]	Retrospective single-arm, multiple-center study	January 2001 and December 2018	IA1- IB2 (FIGO 2018)	176	43.1 ± 11.8	Tc-99m+MB, Tc-99m+ICG, MD, or ICG	Bilateral PLND
Santoro et al. 2020 (Italy) [59]	Retrospective study	January 2018 to March 2020	IA1-IIB (FIGO 2018)	116	Median = 41.0 (range 21.0 to 71.0) for the ultrastaging group; Median = 46.0 (range 33.0 to 87.0) for the OSNA group	ICG	Systematic bilateral PLND
Wang et al. 2020 (China) [60]	Retrospective study	December 2015 to March 2018	IB1-IIA1 (FIGO 2009)	45	45.0 ± 9.8	CNP	PLND
Bjornholt et al. 2021 (Denmark) [61]	Prospective multiple-center study	September 2016 to August 2018	IA1-IIA0 (FIGO 2008)	60	Median = 61.0 (range 24.0 to 85.0)	ICG	Permanent processing with final histological results of the SLN
Diniz et al. 2021 (Brazil) [62]	Retrospective study	May 2014 to April 2020	IA1 (FIGO 2019)	92	Median = 40.0 (range 22.0 to 76.0)	Patent blue or ICG	Systematic PLND
Harano et al. 2021 (Japan) [63]	Prospective one-arm study	January 2009 to January 2021	IA2–IB2 (FIGO 2009)	30	Median = 34.0 (range 23.0 to 40.0)	ICG	Total PLND
Sponholtz et al. 2021 (Denmark) [64]	Multicenter prospective cohort study	March 2017 to January 2021	IA1-IB2 (FIGO 2009)	245	Median = 44.0 (range 26.0 to 84.0)	ICG	Completion PLND
Weissinger et al. 2021 (Germany) [65]	Prospective study	March 2016 to April 2019	IA-IIB (FIGO)	41	48.1 ± 12.2	Tc-99m+ (ICG or blue dye)	Bilateral systematic lymphadenectomy
Ya et al. 2021 (China) [66]	Prospective study	May 2017 to December 2019	(FIGO 2009)	356	Median = 46.0 (range 23.0 to 68.0)	CNP	Complete PLND
Aoki et al. 2022 (Japan) [67]	Prospective single-center cohort study	October 2016 and October 2019	IA2-IB1 (FIGO 2009)	77	Median = 40.0 (range 25.0 to 74.0)	ICG	Systematic PLND
Baeten et al. 2022 (Netherlands) [68]	Prospective, single-center, single-arm feasibility study	Not specified	IA – IB2 or IIA1 (FIGO 2018)	10	Median = 39.0 (range 26.0 to 72.0)	Tc-99m	PLND
Luhrs et al. 2022 (Sweden) [70]	Prospective study	January 2014 to December 2020	IA-IIA (FIGO 2009)	145	Median = 43.6 (range 23.0 to 85.0)	ICG	PLND
Niu et al. 2022 (China) [71]	Prospective study	February 2019 to June 2021	IA-IIB (FIGO)	59	48.6 ± 12.9	Tc-99m	Extensive lymph node dissection
Yahata et al. 2018 (Japan) [49]	Retrospective study	January 2009 to December 2017	IA-IIA (FIGO)	181	Median = 34.0 (range 21.0 to 73.0)	Tc-99m	Final histological results of the SLN
Smits et al. 2023 (United Kingdom) [72]	Retrospective cohort study	October 2015 to October 2019	IA1-IIA1 (FIGO 2009)	100	Median = 39.0 (range 24.0 to 82.0)	ICG	PLND
Amengual et al. 2024 (Spain) [73]	Prospective, observational, descriptive, single-center study	January 2019 to October 2023	IA1-IIA1 (FIGO 2018)	38	Median = 46.5 (range 40.0 to 54.0)	ICG+Tc-99m	Permanent processing with final histological results of the SLN
Bizzarri et al. 2024_ OSNA (Italy) [74]	Single-center, retrospective, cohort study	May 2017 to January 2021	IA-IIA1 (FIGO 2018)	100	Median = 44.0 (range 26.0 to 85.0)	ICG	Bilateral systematic PLND
Bizzarri et al. 2024_ SCCAN (Italy) [75]	International, multicenter, retrospective study	January 2007 to December 2016	IB1-IIA2 (FIGO 2009)	300	37.0% was >45 years	Tc-99m ± blue dye or ICG	Pelvic lymphadenectomy ± para-aortic lymphadenectomy
Bogani et al. 2024 (Europe, Asia, North America, or Latin America) [76]	Retrospective, multi-institutional study	January 2000 to December 2022	IA1-IB1 (FIGO 2009)	31	Median = 33.0 (range 22.0 to 41.0)	Not specified	Bilateral PLND
Persson et al. 2024 (Sweden) [77]	Prospective non-randomized trial	September 2014 to January 2023	IA2-IIA1 (FIGO 2009)	181	Median = 44.5 (range 23.0 to 85.0)	ICG	Complete PLND
Vemula Venkata et al. 2024 (India) [78]	Prospective study	June 2016 to December 2017	IA-IIA (FIGO)	20	Median = 53.0	MB	Complete bilateral PLND

CC: Cervical cancer; CNP: Carbon nanoparticle; FIGO: Federation of Gynecology and Obstetrics; ICG: Indocyanine green; MB: Methylene blue; OSNA: one-step nucleic acid amplification; PLND: Pelvic lymph node dissection; SD: standard deviation; Tc-99m: Technecium-99.

### Risk of bias in studies

The quality assessments of the 49 studies are listed in Table 2. Firstly, most of the studies did not provide information on whether patient selection was consecutive. Furthermore, two studies had inappropriate exclusions [36, 44]. Regarding patient selection, the primary concern was that the studies did not include all clinical stages of ECC. Secondly, for the index test domain, the studies were classified as having ‘unclear’ risk of bias because intraoperative rapid pathological examination or ultrastaging was performed only for the reference standard. However, since the pathological examination procedure was highly consistent with real-world practice, the applicability of the index test for most studies was rated as ‘low risk of bias’. Thirdly, most of the included studies had a low risk of bias in the ‘reference standard’ domain. However, three studies were classified as having a high risk of bias because the conduction of the reference standard was unclear or without systematic bilateral PLND [33, 49, 51]. Finally, only a few studies have unclear risks in the domain of ‘flow and timing’ because there was inadequate information to make a judgment.

**Table 2:**
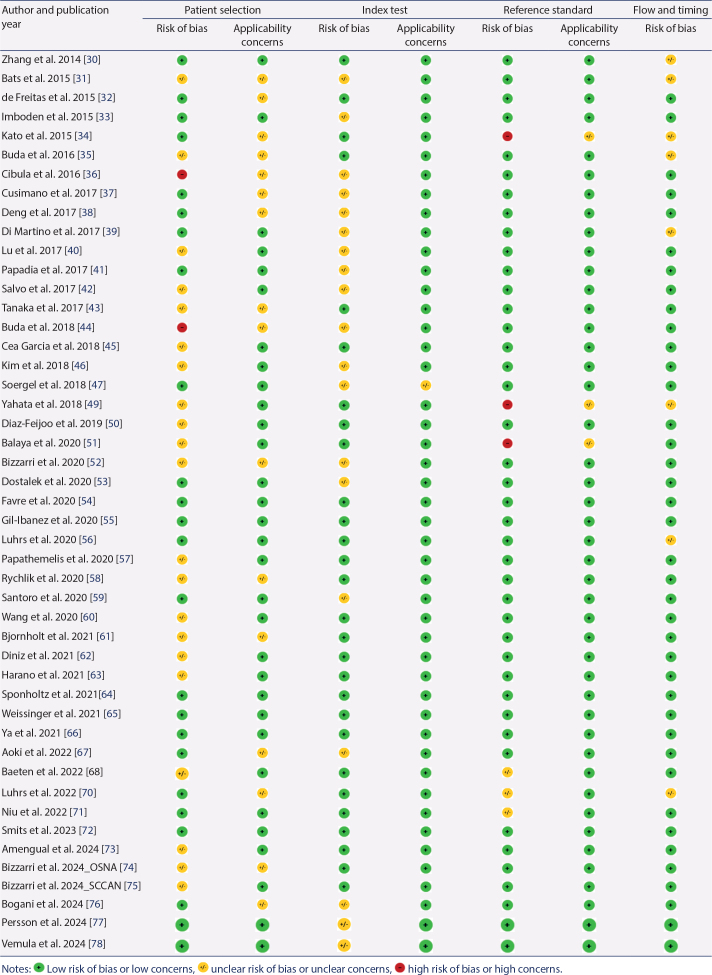
A summary assessment of risk of bias for the 5 included studies using the QUADAS-2 tool.

### Sentinel lymph node false-negative rate

**Figure 2:**
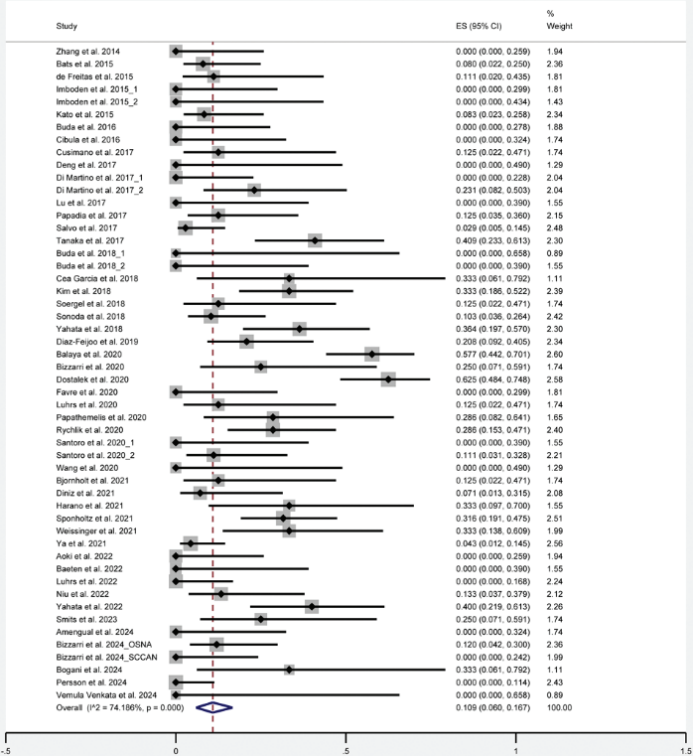
Forest plot of the overall false-negative rate of sentinel lymph nodes for early-stage cervical cancer.

According to the meta-analysis, the FNR of SLNB for ECC was 10.9% (95% confidence interval [CI]: 6.0–16.7) based on 52 data sets from 48 studies (Figure 2). The data of Gil-Ibanez 2020 were excluded from the synthesis as there were no true positive LN metastases in the study [55].

As studies were conducted at different locations, different reference standard methods were applied. For FS using permanent pathological examination of PLND as reference standard, FNR = 18.3% (95% CI: 0.8–45.9); FS using PLND (H&E and ultrastaging), FNR = 8.2% (95% CI: 1.4–17.9); no FS or without considering FS using PLND including SLN (H&E and ultrastaging) as reference standard, FNR = 9.6% (95% CI: 1.8–21.0), one-step nucleic acid amplification (OSNA) instead of FS (H&E) using PLND as reference standard, FNR = 8.3% (95% CI: 1.5–18.3); and FS (H&E) using permanent pathological examination of SLN without PLND as reference standard, FNR = 27.0% (95% CI: 9.2–49.4).

**Figure 3:**
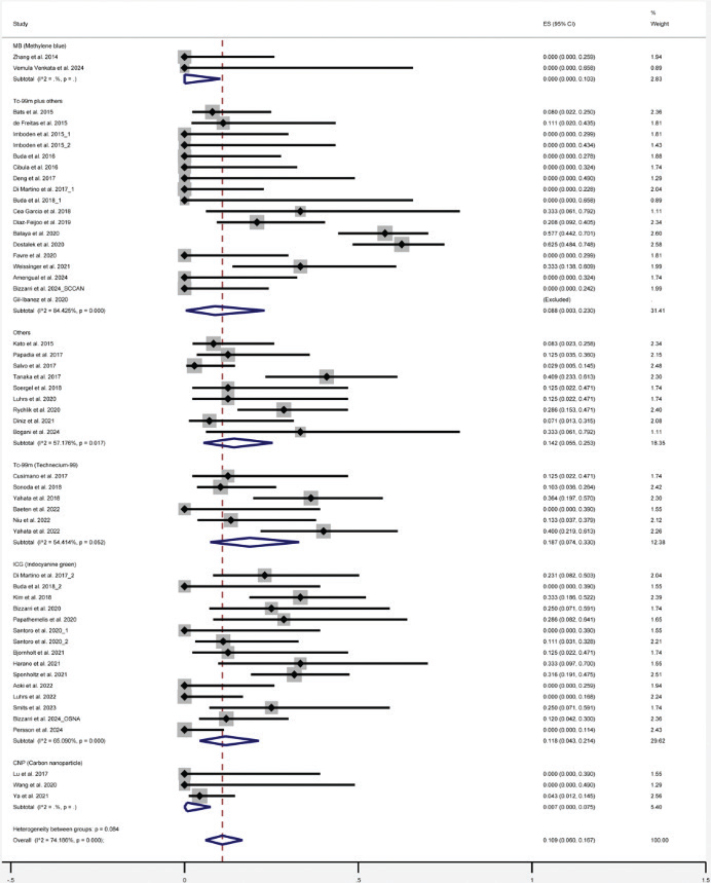
Forest plot of the subgroup analysis of the overall false negative rate of sentinel lymph nodes for early-stage cervical cancer according to different tracer regimens.

There was no significant difference in FNR among patients with ECC across subgroups based on tracer type. The FNRs were 0% (95% CI: 0–10.3), 7.0% (95% CI: 0–7.5), 8.8% (95% CI: 0.3–23.0), 11.8% (95% CI: 4.3–21.4), and 18.7% (95% CI: 7.4–33.0) for methylene blue (MB), carbon nanoparticle (CNP), Tc-99m plus others, indocyanine green (ICG), and Tc-99m alone, respectively (Figure 3).

A total of four studies provided FNR information on tumor diameters ≥ 2 cm and/or < 2 cm [37, 38, 61, 64]. There was no significant difference in FNR in patients with ECC according to tumor diameter. However, the mean FNR (23.1%, 95% CI: 0–61.7) for patients with larger tumor diameters (≥ 2 cm) was higher than that for patients with smaller tumor diameters (< 2 cm) (6.9%, 95% CI: 0–33.1).

### Meta-regression results

According to the results of the meta-regression analysis (Table 3), only the location of the SLN in the obturator area was significantly negatively related to the FNR (coefficient = −0.88, *p* = 0.04). Other variables were not significantly associated with the SLNB FNR in patients with ECC in the meta-analysis. However, age (mean or median), BMI (mean or median), SCC (tumor pathological type, proportion), grade 1 and grade 2 (risk of tumor, proportion), FIGO IA (proportion), FIGO IIA (proportion), SLN located in the para-aortic area (proportion), SLN located in the internal iliac area (proportion), SLN located in the presacral area (proportion), LVSI (proportion), and LEEP (proportion) were found to have a negative association with FNR. On the contrary, AD (portion of tumor pathological type), AS (portion of tumor pathological type), grade 3 (risk of tumor, proportion), FIGO IB (proportion), FIGO IIB (proportion), mean SLN detection number, patient detection rate of SLN, bilateral SLN detection rate, SLN located in the common iliac area (proportion), SLN situated in the external iliac area (proportion), SLN located in the parametrial area (proportion), and patients with neoadjuvant history (proportion) were found to have a positive association with FNR.

**Table 3:** Meta-regression analysis of false-negative rates of sentinel lymph node biopsy for early-stage cervical cancer.

Variables	Contant	Coefficient	*P*-value
Age (mean or median value), years	0.29	−0.003	*P* = 0.434
BMI (mean or median value), kg/m2	0.46	−0.012	*P* = 0.556
SCC, proportion	0.21	−0.11	*P* = 0.558
AD, proportion	0.11	0.11	*P* = 0.531
AS, proportion	0.13	0.13	*P* = 0.800
Grade 1, proportion	0.14	−0.06	*P* = 0.842
Grade 2, proportion	0.18	−0.10	*P* = 0.665
Grade 3, proportion	0.07	0.21	*P* = 0.446
FIGO IA, proportion	0.15	−0.11	*P* = 0.391
FIGO IB, proportion	0.09	0.06	*P* = 0.554
FIGO IIA, proportion	0.15	−0.19	*P* = 0.438
FIGO IIB, proportion	0.13	0.32	*P* = 0.360
SLN number (mean), n	0.11	0.005	*P* = 0.774
SLN detection patients’ rate, %	0.07	0.09	*P* = 0.147
Bilateral SLN patients’ rate, %	0.05	0.13	*P* = 0.232
Para-aortic (SLN location), proportion	0.14	−0.88	*P* = 0.701
Common iliac (SLN location), proportion	0.11	0.21	*P* = 0.701
External iliac (SLN location), proportion	0.05	0.23	*P* = 0.177
Internal iliac (SLN location), proportion	0.15	−0.53	*P* = 0.389
Obturator (SLN location), proportion	0.27	−0.34	*P* = 0.04
Presacral (SLN location), proportion	0.14	−1.06	*P* = 0.692
Parametrial (SLN location), proportion	0.099	1.68	*P* = 0.379
LVSI, proportion	0.18	−0.74	*P* = 0.768
LEEP, proportion	0.20	−0.08	*P* = 0.677
Neoadjuvant, proportion	0.13	0.63	*P* = 0.201

AD: Adenocarcinoma; AS: Adenosquamous cell carcinoma; BMI: body mass index; FIGO: the International Federation of Gynecology and Obstetrics; LEEP: Loop electrosurgical excision procedure; LVSI: lymphovascular space invasion; SCC: Squamous cell carcinoma; SLN: sentinel lymph node.

### Sensitivity analysis and reporting biases

**Figure 4:**
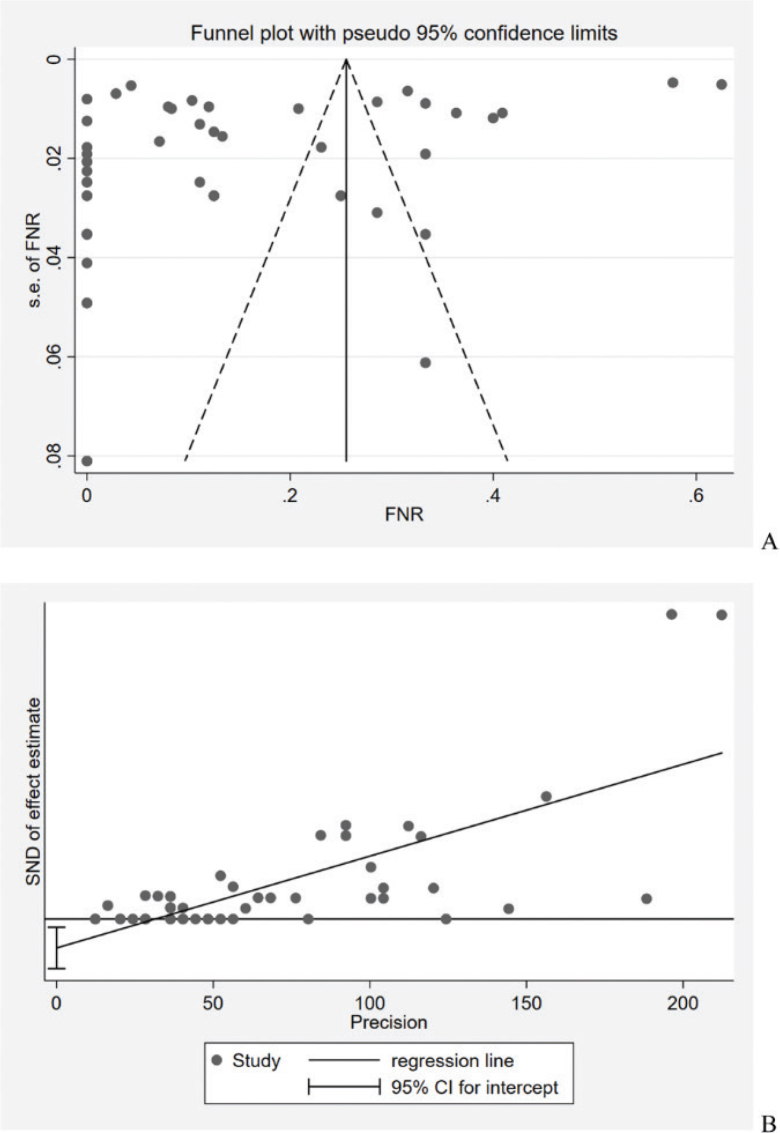
Publication bias assessment. (A) Funnel plots of false negative rates in the included studies. (B) Egger test for small-study effects on false-negative rates in the included studies.

The FNA result was 9.8% (95% CI: 4.8–15.7) by sensitivity analysis, which is similar to the original result. According to the funnel plots and Egger’s test for small-study effects (*p* = 0.007) illustrated in Figure 4, there was no publication bias in this study.

## Discussion

In this study, we performed a meta-analysis of 49 articles involving 5,163 patients with ECC who underwent SLN studies. The study quality and clinical applicability of the included studies had a low to moderate risk of concern. According to our analysis, the overall FNR of SLNB for ECC was 10.9% (95% CI: 6.0–16.7). Next, no significant differences in FNR were found for different reference standards or tumor diameter (< 2 cm vs. ≥ 2 cm). However, different tracers appear to account for different FNRs. In the meta-analysis, we found that the proportion of SLN located in the obturator area was significantly negatively associated with FNR (coefficient = −0.88, *p* = 0.04).

Based on studies published between 2014 and 2024, we found that the overall FNR of SLNB for ECC was 10.9% (95% CI: 6.0–16.7), which is remarkably similar to existing evidence. Lai et al. conducted a systematic review without meta-analysis and reported an FNR of 9% based on studies published between 1999 and 2015 [79] and Frumovitz et al. reported an FNR of more than 8% based on studies published from 2000 to 2007 [80]. Based on those results, FNR is a continuing issue for the surgical treatment of ECC, which surgeons must keep in mind when performing SLNB for ECC.

A critical controversy regarding SLNB is whether LVM (including micrometastases [MIC] and isolated tumor cells [ITC]) can be detected by intraoperative pathological techniques [81]. However, the traditional H&E technique used for intraoperative FSs cannot detect LVM, and IHC ultrastaging is believed to be the first choice to fulfill this purpose [17, 82, 83]. Another intraoperative LVM detection method is OSNA, which showed that patients using OSNA were not associated with worse DFS compared to those using ultrastaging [74]. Rationally, if the ultrastaging technique or OSNA were applied during the SLN assessment, the FNR should be lower than that of those who did not. However, according to our FNR subgroup analysis, which uses different techniques for the SLN test, no statistically significant differences were found. Our results may be due to the small sample size of patients included for different techniques or other potential risk factors. However, according to a recent meta-analysis by Guani et al., the risk ratios for disease-free survival (DFS) and overall survival (OS) in patients with LVM (MIC + ITC) compared to nonmetastatic patients were 2.60 (95% CI: 1.55–4.34) and 5.65 (2.81–11.39), respectively [84]. Therefore, in practice, it is never prudent to omit the appropriate techniques for detecting LVM in SLNB.

The tracers used during surgery were believed to be one of the key influencers for the precision of SLNB [16]. Both in breast cancer and CC, ICG was reported to have a better SLN detection rate than other tracers [16, 85]. Similar reports indicate that Tc-99m combined with other tracers was better than Tc-99m alone [35, 86]. According to our subgroup analysis of FNR, the means of the different tracer regimens were consistent with the above SLN detection rate. However, this difference was not statistically significant. Furthermore, only one study with a small sample size (*n* = 20) used MB as a tracer. More high-quality studies are needed to verify the accuracy of the different tracers used for SLNB.

In this study, four studies provided FNR data for different tumor diameters (≥ 2 cm vs. < 2 cm) [37, 38, 61, 64]. There was no significant difference in FNR in patients with ECC according to tumor diameter. However, the mean FNR for patients with larger tumor diameters (≥ 2 cm) was higher than that for patients with smaller tumor diameters (< 2 cm). Our findings were consistent with those reported by Zhang et al. [87]. Although more evidence is required to confirm this, in practice, more attention should be paid to the FNR issue when ECC tumor diameters are > 2 cm.

Limited by study-level data, we identified a few risk factors that could statistically affect the FNR. The only factor found was the proportion of SLN located in the obturator fossa, which was negatively associated with FNR. The obturator area is one of the most common sites for SLN identification. Our results indicated that the more SLNs found in the common SLN area, the fewer FNRs found. However, there are no similar reports in the literature on this topic. Whether SLN’s location is associated with the accuracy of the SLBM needs to be confirmed in future research.

This study is the first systematic review and meta-analysis of SLNB for ECC. In addition, it is the first to explore FNR risk factors using subgroup analysis and meta-regression. Our study has the advantage of a larger number of articles and a more recent publication year (in the last decade), allowing us to provide a more comprehensive and up-to-date summary of the topic of SLNB FNR for ECC. However, due to limited patient-level data, we identified only a few statistically significant risk factors for FNR. Furthermore, we provided evidence of trends in this topic that offer valuable clues for future research. The next limitation of this study is that the quality of the evidence may be low due to data heterogeneity. Future clinical trials following the standard procedures are needed to confirm or clarify our findings.

## Conclusions

The overall FNR of SLNB for early CC was 10.9% (95% CI: 6.0–16.7). Factors that tended to reduce the FNR included the use of ultrastaging or OSNA for LVM detection, having a tumor diameter of less than 2 cm, employing specific tracer regimens (e.g. ICG, CNP, and Tc-99m combined with other tracers), and identification of more than one lymph node in the obturator fossa. More research is needed to confirm this hypothesis.

## Supplementary Material



## Data Availability

The datasets generated and analyzed during this study are available from the corresponding author on reasonable request.
